# Differentially expressed exosome miRNA profiles as putative prognostic biomarkers for profound sudden sensorineural hearing loss

**DOI:** 10.1186/s12920-026-02330-9

**Published:** 2026-02-24

**Authors:** Hongru Zhang, Haoliang Xie, Qiran Cao, Ping Chen, Sen Yan, Xin Zhang, Pei Gao, Yu Zhang

**Affiliations:** 1 Department of Otolaryngology, Beijing Haidian Hospital, Beijing, 100080 People’s Republic of China; 2 School of Basic Medical Sciences, Capital Medical University, Beijing, 100069 People’s Republic of China; 3 Thurgood Marshall College, University of California, San Diego, La Jolla, San Diego, CA 92092-2002 USA

**Keywords:** Profound SSNHL, Exosomes MicroRNA, sRNA sequencing, Prognosis

## Abstract

**Background:**

Sudden sensorineural hearing loss (SSNHL) is an abrupt, often idiopathic hearing decline with uncertain prognosis and multifactorial pathophysiology. Among its subtypes, total deafness—also referred to as profound SSNHL—carries the worst and most unpredictable prognosis, highlighting an urgent need for reliable biomarkers, especially for prognostic biomarkers. Recent studies have emphasized the potential of exosome microRNAs (miRNAs) as both biomarkers and therapeutic targets in SSNHL, yet very few studies have focused on the profound type. This study aims to preliminarily identify the plasma-derived exosome miRNAs as candidate biomarkers, particularly possible prognostic indicators, in patients with profound SSNHL.

**Methods:**

Of 32 patients with profound SSNHL, six who achieved complete recovery and six age-matched patients with no improvement after two weeks of glucocorticoid therapy were selected for this retrospective study. Six age-matched healthy individuals with normal hearing served as controls. Pretreatment plasma samples were collected, exosomes purified and stored at -80℃. The exosome samples from 18 enrolled participants were subjected to RNA extraction and small RNA sequencing. Differentially expressed exosome miRNAs (DEEMs) were identified using the DESeq R package, with significance thresholds set at *p* < 0.05 and |log₂(fold change)| > 1. Predicted target mRNAs of DEEMs between the recovery and no improvement groups were analyzed for pathway enrichment using the Metascape database.

**Results:**

We identified 12 candidate DEEMs (10 downregulated and 2 upregulated) shared across all profound SSNHL groups compared to healthy controls, and 14 candidate DEEMs (6 downregulated and 8 upregulated) between the no improvement group and the recovery group. The six pronounced changed miRNAs out of those 14 were hsa-miR-320d, hsa-miR-146a-3p, hsa-miR-132-3p, hsa-miR-9-3p, hsa-miR-219b-5p, and hsa-miR-219a-2-3p. The target mRNAs of these top six miRNAs were mainly enriched in pathways of embryonic development, tube morphogenesis, cell division, synaptic transmission, STAT3 and TGF-β signaling, autophagy, cellular import, ketone response, and cellular responses to interleukin-1 and stress.

**Conclusions:**

By exosomal miRNA profiling from patients of different treatment outcomes,, this exploratory study initially identified potential DEEMs that may serve as candidate biomarkers for profound SSNHL, although larger, independently replicated studies are required.

**Supplementary Information:**

The online version contains supplementary material available at 10.1186/s12920-026-02330-9.

## Background

Sudden sensorineural hearing loss (SSNHL) is a clinical emergency characterized by rapid, unexplained sensorineural hearing loss of 30 dB or greater over at least three contiguous audiometric frequencies occurring within a 72-hour period, and exhibits a high incidence globally [1]. SSNHL is sub-classified into four phenotypes; low-frequency descending type, high-frequency descending type, flat type (all-frequency descent) and total-deafness type (profound or “deaf” curve), defined by the pattern of threshold shift on the initial pure-tone audiogram [2]. Among them, profound SSNHL carries the worst and unpredictable prognosis; fewer than 10–15% of patients recover any serviceable hearing, while more than 50%-60% of patients obtain no improvement of hearing loss after the mainstay of steroid therapy [3–5]. Identifying the potential causes and characterizing the underlying molecular mechanisms therefore is of great importance to formulate treatment plans, predict and improve prognosis as well.

MicroRNAs (miRNAs) are endogenous, ~ 22-nucleotide-long non-coding RNAs that silence gene expression through sequence-specific binding to target mRNAs, resulting in translational repression or transcript degradation. Accumulating molecular evidence has implicated discrete miRNA networks in the pathogenesis of acquired hearing loss by governing auditory cell survival, inflammatory signaling, hypoxia adaptation, and cochlear vascular homeostasis [6–9].

Exosomes—30–150 nm lipid-bilayer extracellular vesicles secreted by virtually all cell types—shield their RNA cargo from ribonuclease activity and fluctuating extracellular conditions, thereby preserving biological information and enabling intercellular communication via ligand–receptor interactions or direct cargo delivery [10, 11]. In recent years, exosome-encapsulated miRNAs have been increasingly recognized as both robust indicators of disease status and modulators of therapeutic responses. For example, serum miRNAs are suspected to be closely related to SSNHL pathogenesis or serve as biomarkers for SSNHL [12]. And plasma extracellular-vesicle (EV)-derived complement C3 is reported to correlate with baseline severity and early steroid responsiveness in patients with profound SSNHL [13]. Nevertheless, the clinical value of differentially expressed exosome miRNAs (DEEMs) as prognostic determinants for profound SSNHL remains unexplored.

This exploratory study aims to identify candidate DDEMs in plasma samples from profound SSNHL patients, in particular from patient of different outcomes after 2 weeks’ standard glucocorticoid therapy.

## Methods

### Study populations and diagnosis

Adult patients, aged 18–65, presenting with sudden hearing loss(onset within 72 h)and received no prior treatment were recruited consecutively at the Department of Otolaryngology, Beijing Haidian Hospital from May 2019 to September 2020. Profound-type sudden sensorineural hearing loss (total-deafness subtype) was defined as a four-frequency (500, 1000, 2000, 4000 Hz) pure-tone average ≥ 81 dB HL in the affected ear by pure-tone audiometry (PTA), according to the WHO-ICIDH classification of hearing impairment [14].

Inclusion/exclusion criteria is as follows: those with sudden hearing loss in one or both ears within minutes to 3 days and met diagnosis of total deafness were included. Those with contraindications for medications to hormones or defibrinogenation were excluded. Those with an identified cause of hearing loss, major medical illness, or coexisting ear pathology were excluded according to otoscopic or inner ear MRI examinations, tympanometry with acoustic reflex testing, and the tuning fork tests. Exclude pregnant women. Recruited patients hospitalized and received a comprehensive treatment strategy combining glucocorticoid therapy, antifibrinolytic therapy, and neurotrophic support. Patients admitted to the otorhinolaryngology department for surgical treatment of nasal septal deviation volunteered to this study as healthy hearing controls during the same period. The plasma exosome samples from the recruited participants were extracted and stocked as described below.

After 2 weeks of standardized inpatient therapy, hearing recovery for those profound SSNHL was assessed with four-frequency PTA and graded according to the modified Siegel criteria as: complete recovery (final PTA ≤ 10 dB HL of the contralateral ear), marked improvement (PTA gain ≥ 30 dB but not reaching complete recovery), slight improvement (PTA gain 15–29 dB) and no improvement (PTA gain < 15 dB) [15]. In this study, we kept all cases in a complete recovery group and selected equal numbers of age- and sex-matched participants from both the no improvement group and the healthy hearing control group to explore the possible prognostic factors.

## Audiological testing

All otoscopic and audiological procedures were performed by study-certified clinical audiologists. Pure-tone air-conduction thresholds were measured at 250, 500, 1 000, 2 000, 4 000 and 8 000 Hz; bone-conduction thresholds were recorded when the air-conduction gap ≥ 10 dB. Middle-ear function was evaluated with 226-Hz tympanometry and ipsilateral/contralateral acoustic reflexes. Auditory brain-stem response (ABR), auditory steady-state response (ASSR), and distortion-product otoacoustic emissions (DPOAEs) were obtained as needed to confirm cochlear site-of-lesion and to estimate thresholds when behavioural responses were unreliable. All data were extracted from the electronic medical record and cross-validated by a trained reviewer panel.

### Blood sample collection for laboratory test and MicroRNA sequencing

Blood samples from the subjects were collected in separate tubes designated for serum and plasma separation within 3 days of admission to the hospital. Serum and plasma aliquots were subject to laboratory testing, including blood routine, coagulation function, liver function, and infectious or immune related disease antibodies. For exosome sRNA extraction, 10 ml blood was collected from each participant into tubes containing 3.2% sodium citrate as an anticoagulant (0.2 mL per tube), followed by centrifugation at 4,000 rpm for 10 min at 4 °C, and the supernatant exosome in plasma was transferred into cryogenic vials and sent to Novogene (Beijing, China) for sRNA extraction, quantification, qualification, library preparation and sequencing.

### RNA isolation, library preparation, and sRNA sequencing

sRNA Library construction was performed using standard NEB library preparation protocols and sequenced with an Illumina HiSeq 2500/2000 (SE150; Novogene) [16]. Briefly, total RNA was extracted and purified using RNeasy Mini Kit with on-column DNase digestion (Qiagen). RNA purity and integrity were tested by nanodrop, gel electrophoresis, and in the Agilent 2100 bioanalyzer. The sequencing libraries were generated using the NEB Next Multiplex Small RNA Library Prep Set for Illumina, following the manufacturer’s recommendations, and index codes were added to attribute sequences to each sample (Novogene). The first strand cDNA was synthesized using M-MuLV Reverse Transcriptase (RNase H–) and amplified with PCR using LongAmp Taq 2X Master Mix, SR Primer for Illumina, and index(X)primer. PCR products were purified on 8% polyacrylamide gel, and DNA fragments of 140 ~ 160 bp in length were recovered. The library quality, the insert size, was assessed on the Agilent Bioanalyzer 2100 system using DNA High Sensitivity Chips. The clustering and sequencing of the index-coded samples was performed on a cBot Cluster Generation System using TruSeqSR Cluster Kit v3-cBot-HS (Illumia) and on an Illumina Hiseq 2500/2000 platform, respectively, and 50 bp single-end reads (SE50) were generated.

### Bioinformatics analysis/ data analysis

After quality control, the raw data was processed to obtain clean data (clean reads) through custom perl and python scripts, and sRNA of 18 ~ 35nt length from clean reads was chosen to conduct the downstream analyses, including known miRNA alignment, novel miRNA prediction, miRNA quantification, target gene prediction, and Gene Ontology and Kyoto Encyclopedia of Genes and Genomes (GO and KEGG) enrichment analysis. In this study, we focus on differentially expressed miRNAs and their predicted target mRNAs for their involvement in biological pathways relevant to SSNHL pathogenesis, especially for the profound type. Briefly, the small RNA tags were mapped to the reference sequence by Bowtie without mismatches to analyze their expression and distribution on the reference. Mapped small RNA tags were used to look for known miRNA using miRBase20.0 as a reference. miRNA expression levels were estimated by TPM (transcript per million) through the following normalization formula: Normalized expression = mapped readcount/Total reads*1,000,000. Predicting the target gene of miRNA was performed by psRobot_tar in miRanda and RNAhybrid [17, 18]. The predicted target genes of the DEEMs of interest were put togerther for the pathway and process enrichment analysis using the Metascape database (www.metascape.org) (v3.5.20250701) for annotation and visualization.

### Statistical analysis

Student t test or Mann-Whitney U test was performed to compare the continuous variables, which were summarized as means ± standard deviations or medians (interquartile ranges, IQR), whereas the Chi-square test and Fisher’s exact test were used for comparing categorical variables, which were presented as number (percentage), as appropriate. All statistical tests were two-sided, and a P-value less than 0.05 was defined as statistically significant. Data were analyzed using SAS 9.4 (SAS Institute, Cary, NC).

The differential expression of miRNA was performed using the DESeq R package (3.0.3). The p-value < 0.05 and |log2(foldchange)|>1 were set as the threshold for significance and run further analysis, such as clustering in samples and target mRNAs pathway enrichment etc. For the biological functions and signaling pathways enrichment analysis, all genes in the genome have been used as the enrichment background. Terms with p-value < 0.01, a minimum count of 3, and an enrichment factor > 1.5 (the enrichment factor is the ratio between the observed counts and the counts expected by chance) are collected and grouped into clusters based on their membership similarities. The most statistically significant term within a cluster is chosen to represent the cluster. The statistical test values of GOKEGG were all processed with -log10(value) for the ranking of reliable terms.

## Results

### Study populations and laboratory findings

**Fig. 1:**
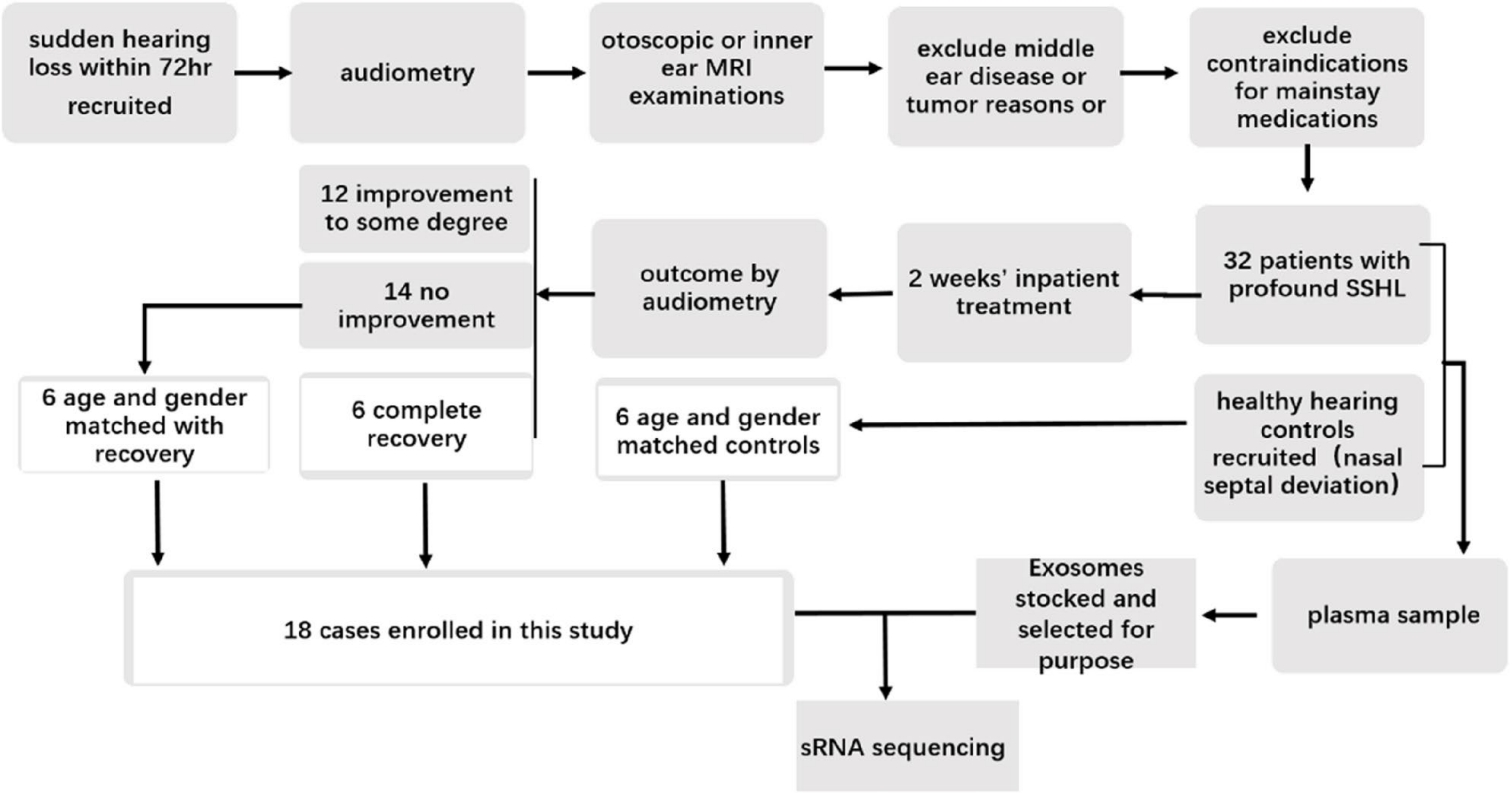
Flow diagram of patient charts that were included and excluded in our analysis. 32 patients diagnosed as profound SSNHL were recruited. After two weeks’ treatment, 6 patients obtained complete recovery, 14 patients showed no improvement, and 12 patients showed improvement to some degree. After age and gender-match, 6 recovery patients and 6 no improvement patients were enrolled in this study, together with 6 matched healthy hearing controls. Accordingly, their pretreatment plasma exosomes were subject to sRNA sequencing, and laboratory test records were extracted

In this study, 32 profound SSNHL patients who met diagnostic criteria of mean threshold ≥ 81 dB HL at four frequencies (500, 1000, 2000, 4000 Hz) in pure-tone audiometry were recruited and received systematic glucocorticoid therapy, combined with antifibrinolytic therapy and neurotrophic support. Two weeks later, hearing recovery was evaluated according to the PTA record. Among those 32 patients, 6 patients obtained complete recovery, with mean audiometric thresholds decreased from 90 dB to 30 dB, 14 patients showed no improvement, and 12 patients showed improvement to some degree. Six patients who are age and gender-matched with the 6 recovery patients were selected among the 14 who showed no improvement. Among 20 healthy hearing volunteers, six age and gender-matched patients admitted to the Otorhinolaryngology (ENT) department for surgical treatment of nasal septal deviation were selected as paired controls. In total, 12 profound SSNHL patients (6 of complete recovery and 6 of no improvement) and 6 healthy hearing controls are included in this study (Fig. 1).

As summarized in supplementary Table 1, the primary symptoms in the deafness group were tinnitus, vertigo, and aural fullness, with the majority of patients presenting to the emergency department. Some laboratory parameters showed differences between total deafness and the healthy hearing controls, although all values remained within the normal range (supplementary Table 2). For example, urea level and uric acid (UA) level were lower in profound SSNHL patients compared to controls, while carbon dioxide combining power (CO₂CP) were higher in SSNHL patients compared to controls. Despite the limited number of cases and the normal range of those index, there might hint a trend toward metabolic abnormalities in SSNHL-TD patients, which requires further research in the future.

Regarding outcomes, demographic features revealed that the complete recovery group generally sought medical attention earlier after symptom onset, with an average onset-to-treatment time of 0.5 days versus 1 day in the no improvement group (supplementary Table 3), and no laboratory findings of particular clinical significance were identified between the two groups (supplementary Table 4).

### Differential expression of exosome MiRNAs in profound SSNHL and in patients of poor prognosis

sRNA sequencing was performed to identify differentially expressed miRNAs in plasma-derived exosomes from pretreatment patients. As 12 profound SSNHL patients are subgrouped into 6 recovered and 6 no improvement, the samples were analyzed for hearing loss group (before treatment, BT), recovered (BR), and no improvement (BN) in comparison to healthy hearing controls (HC), as well as the comparison between BR and BN.

**Fig. 2:**
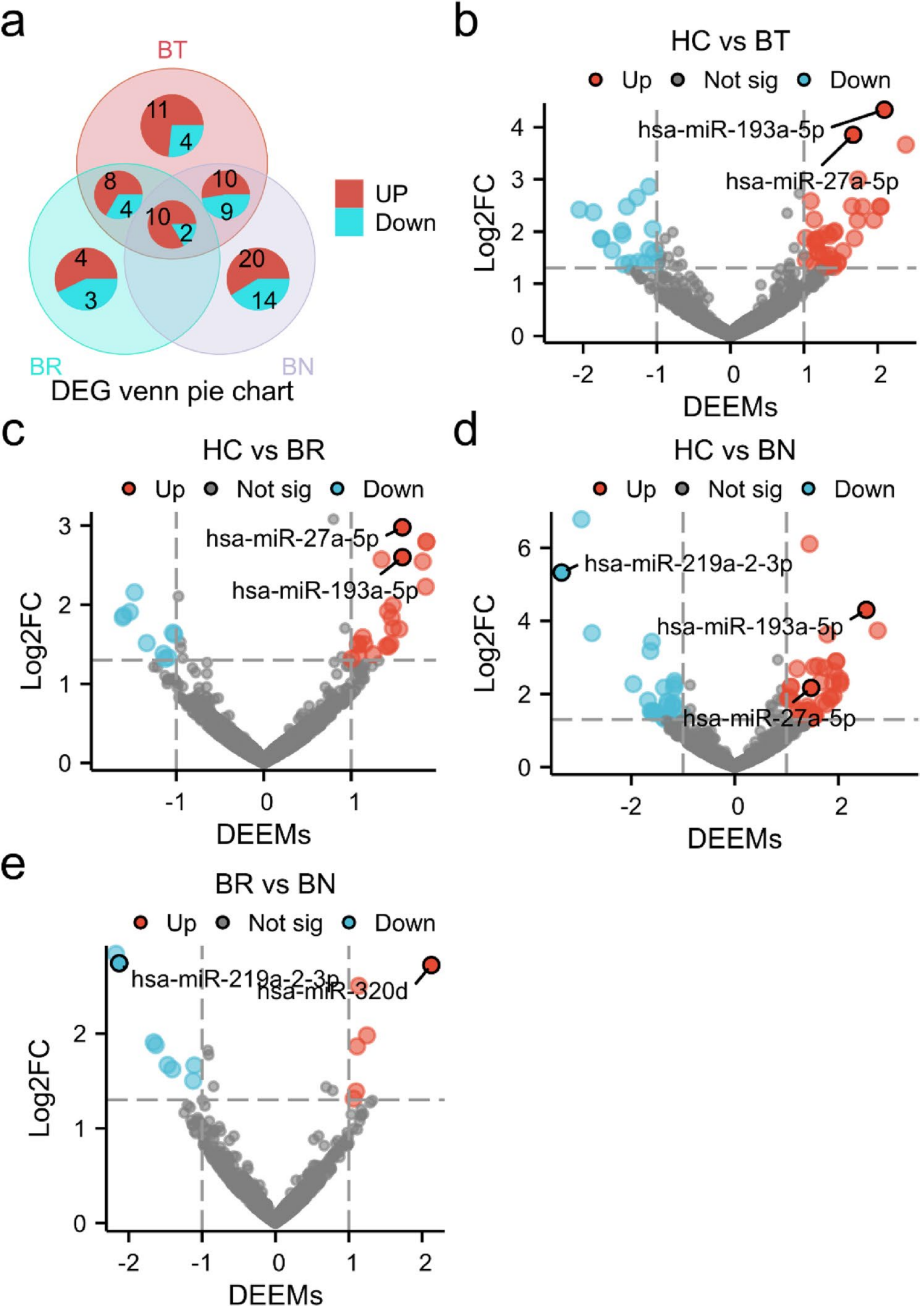
The distribution and profiling of DEEMs identified in profound SSNHL group and across prognostic subgroups. **a** Distribution of DEEMs between pretreatment samples visualized in venn pie graph. Numbers of up- (red) and down-regulated (navy blue) miRNAs in each comparison with control are shown. **b**-**e** Volcanic of differential miRNAs between indicated groups. red represented the up-regulated miRNA while green represented the down-regulated miRNA with significance thresholds set at p < 0.05 and |log₂(fold change)| > 1. The horizontal line indicates p = 0.05. BT, before treatment hearing loss group; BR, before treatment sample from recovered group; BN, before treatment sample from no improvement group; HC, healthy hearing controls

In comparison to healthy controls, the distribution and profiling of deferentially expressed candidate miRNAs in profound SSNHL groups was identified and visualized in venn pie graph (Fig. 2a) and vlcano plot (Fig. 2b-d). We found that 58 differentially expressed candidate miRNAs, with 39 downregulated and 19 upregulated, were identified in profound SSNHL (total deafness) patients. A total of 31 differentially expressed miRNAs (22 downregulated and 9 upregulated) were identified in samples of BR group, while 65 miRNAs (40 downregulated and 25 upregulated) were identified in BN group. Among those DEEMs in comparison to healthy hearing controls, 12 miRNAs were shared by all 3 groups (herein after shared miRNAs), implying they are candidates of differentially expressed regardless of treatment outcomes. When comparing between prognostic subgroups (BI with BR), we identified a total of 14 differentially expressed candidate miRNAs (herein after possible prognostic miRNAs or unique miRNAs) (Fig. 2e).

**Fig. 3:**
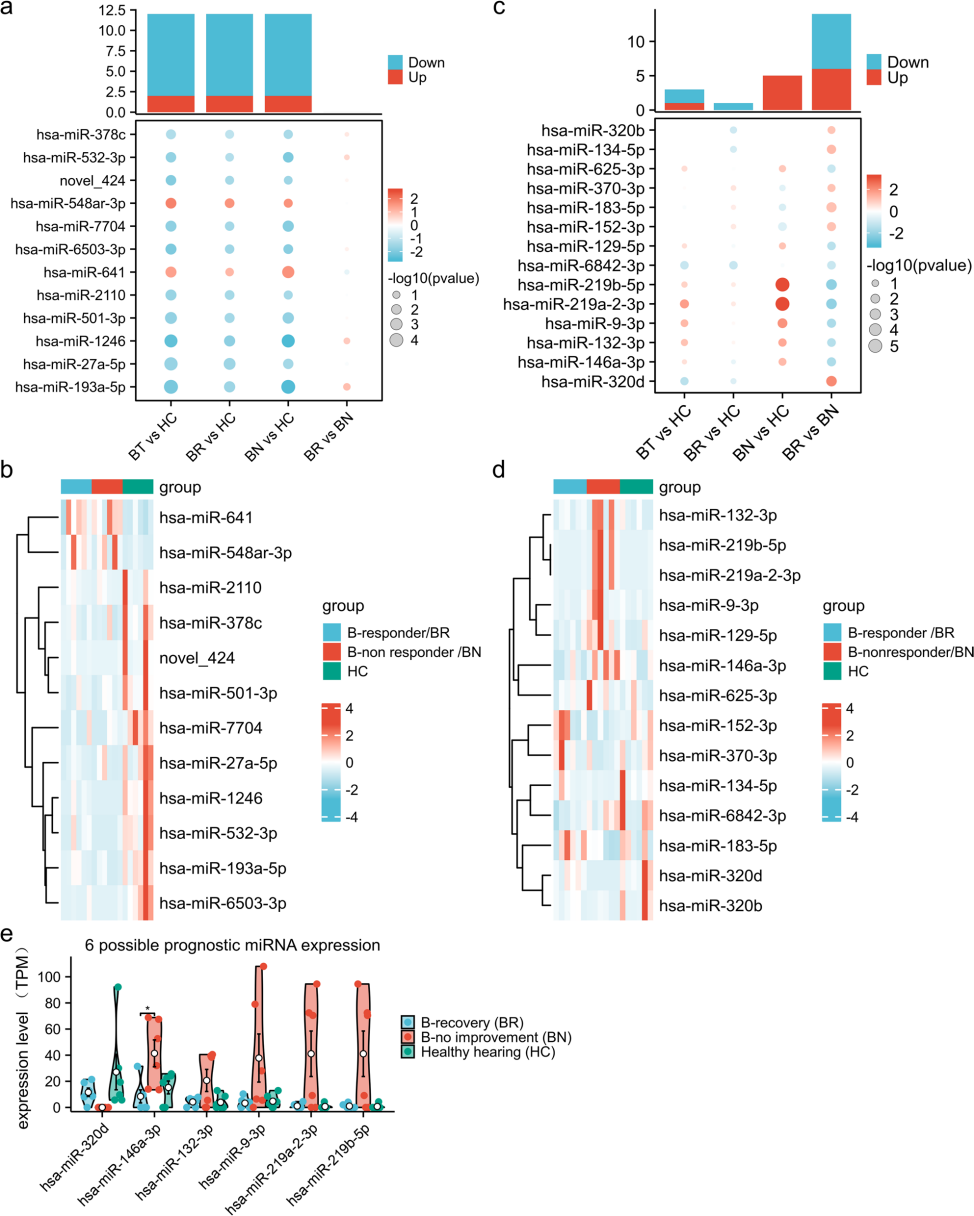
Combined heatmap of differential miRNAs by pairwise comparisons among the four groups and in individuals of each group. **a**, **b** The dot heatmap and clusterd heapmap of 12 shared dysregulated miRNAs. **c**, **d** The dot heatmap and clusterd heapmap of 14 prognostic miRNAs. **e** The relative expression (TPM) of top 6 changed prognostic miRNAs in each group by violin plots. In a and c, the column chart indicate numbers of significantly up- (red) and down-regulated (navy blue) miRNAs in each comparison, and the lower dot heatmap displayed the relative expression of each indicated miRNA in each comparison where dot colour represented log2FC and size indicated –log 10(pvalue)(**a**, **c**). In the cluster heatmap (**b**,**d**), rows (miRNAs) and columns (samples) were clustered according expression profiles

The candidate DEE miRNAs of both 12 shared (10 downregulated and 2 upregulated) in all hearing loss groups and 14 unique (8 downregulated and 6 upregulated) in the recovery group versus the no improvement group were summarized (Table 1). The express pattern in the different patient groups as well as in individual samples of these miRNAs, was also visualized by heat maps and clustering (Fig. 3a-d) to compare and contrast the significant associations. The relative expression of top 6 pronounced changed miRNAs, including hsa-miR-320d, hsa-miR-146a-3p, hsa-miR-132-3p, hsa-miR-9-3p, has-miR-219b-5p and hsa-miR-219a-2-3p, were visualized in violin plots (Fig. 3e), illustrating possible relevance to outcomes for profound SSNHL patients.

**Table 1:** The summary of both shared and unique DEE MiRNAs across prognostic sub-groups

	sRNA	BT vs. HC		BR vs. HC		BN vs. HC		BR vs. BN	
		log2FC	pval	log2FC	pval	log2FC	pval	log2FC	pval
shared miRNA in B, BR and BN	hsa-miR-193a-5p	-2.0922	4.62E-05	-1.5867	0.0025028	-2.5416	4.98E-05	0.97899	0.11077
	hsa-miR-27a-5p	-1.668	0.00014	-1.5852	0.0010506	-1.4759	0.006782	-0.12312	0.78262
	hsa-miR-1246	-2.3833	0.000215	-1.8188	0.0028384	-2.761	0.000183	0.79192	0.18948
	hsa-miR-501-3p	-1.7335	0.000994	-1.4753	0.010147	-1.7395	0.00193	0.094329	0.87924
	hsa-miR-2110	-1.6528	0.003265	-1.4613	0.014484	-1.4436	0.027533	-0.063868	0.91136
	hsa-miR-641	1.4063	0.00333	1.0897	0.046261	1.6312	0.000666	-0.42039	0.44878
	hsa-miR-6503-3p	-2.0312	0.003348	-1.5497	0.020215	-1.8558	0.013464	0.2107	0.68281
	hsa-miR-7704	-1.7955	0.003389	-1.4771	0.019788	-1.9154	0.002447	0.010389	NA
	hsa-miR-548ar-3p	1.8613	0.004258	1.6109	0.014538	1.6259	0.029365	-0.07456	0.90932
	novel_424	-1.9502	0.006001	-1.4499	0.031221	-1.557	0.038314	0.15181	0.76577
	hsa-miR-532-3p	-1.7288	0.006113	-1.2501	0.042186	-2.0326	0.005395	0.60284	0.35537
	hsa-miR-378c	-1.3029	0.010971	-1.0764	0.032186	-1.4022	0.021873	0.34597	0.56661
differentially expressed in BR vs. BN	hsa-miR-320d	-1.3865	0.033693	-0.71874	0.23685	-2.75	NA	2.1254	0.001895
	hsa-miR-146a-3p	0.54864	0.41498	-0.46191	0.49247	1.2589	0.047811	-1.6313	0.013253
	hsa-miR-132-3p	1.1006	0.09994	0.19039	0.77652	1.5682	0.03145	-1.4697	0.021453
	hsa-miR-9-3p	1.2828	0.05837	0.13127	0.84471	1.9615	0.00534	-1.6586	0.01236
	hsa-miR-219a-2-3p	1.7589	0.013641	0.39098	0.49111	3.3471	4.72E-06	-2.1293	0.001812
	hsa-miR-219b-5p	0.81079	0.19706	0.37079	0.51198	3.3471	4.72E-06	-2.1735	0.001442
	hsa-miR-6842-3p	-1.1326	0.025846	-1.1326	0.025846	-0.14665	0.76131	-1.1053	0.021675
	hsa-miR-129-5p	0.5618	0.3911	-0.19496	0.77016	1.1029	0.10874	-1.406	0.023796
	hsa-miR-134-5p	-1.4259	NA	-0.86736	0.12283	-2.1228	NA	1.2472	0.010453
	hsa-miR-152-3p	-0.0058085	0.98821	0.40247	0.34057	-0.69531	0.020154	1.1116	0.01365
	hsa-miR-183-5p	-0.1397	0.73649	0.33835	0.41743	-0.78622	0.08753	1.1353	0.003137
	hsa-miR-320b	-1.577	NA	-0.9948	0.097279	-2.0988	NA	1.0675	0.048569
	hsa-miR-370-3p	0.066076	0.89122	0.49195	0.32048	-0.61439	0.18522	1.0989	0.040828
	hsa-miR-625-3p	0.61396	0.24924	-0.06082	0.91274	1.0045	0.092472	-1.121	0.031459

Only miRNAs shared across all groups or unique miRNAs between recovery and no improvement with |log₂FC| ≥ 1 and pvalue < 0.05 are listed. log₂FC: log₂ fold-change of the first-named group versus the second-named group; positive values indicate up-regulation in the first group, negative values indicate down-regulation. P-value: raw p-value from DESeq2 Wald test

*BT* Before treatment hearing loss group, *BR * Before treatment sample from recovered group，*BN* Before treatment sample from no improvement group, *HC* Healthy hearing controls

### Predicted target mRNAs and enriched pathways

**Fig. 4:**
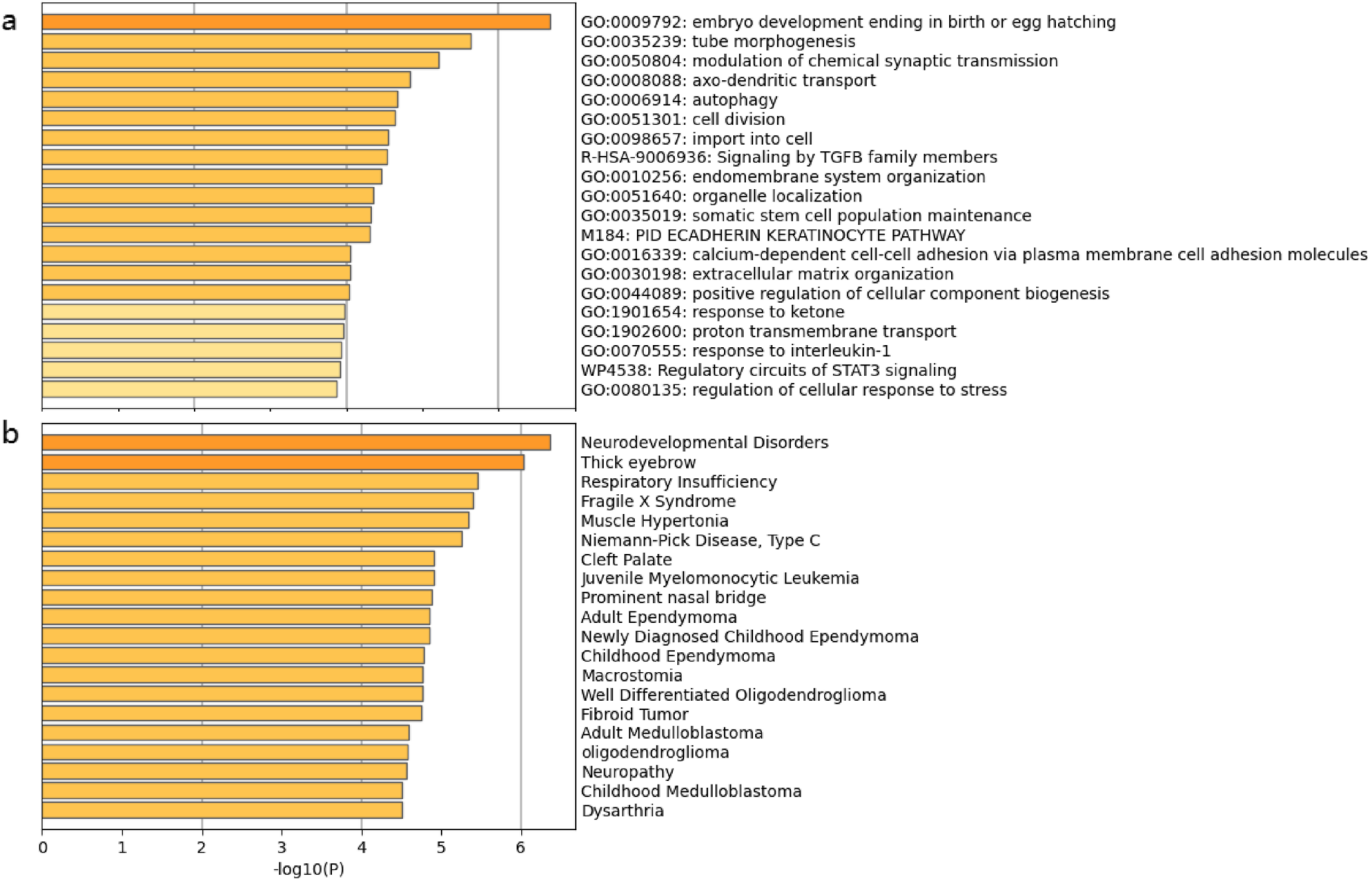
The bar graph of enriched terms across 298 predicted targeted genes for the top 6 prognostic miRNAs using Metascape (**a**) or in DisGeNET (**b**). The top 20 clusters were shown with colored by p-values

A total of 298 potential target mRNAs for the top 6 candidate DEE miRNAs between recovery and nonresponsive groups have been predicted and processed to pathway and GO/KEGG enrichment analysis to identify potential pathways involved in outcomes for heavy hearing loss in SSNHL. Among the relative top 20 pathways, the target mRNA is mainly enriched in embryo development, tube morphogenesis, cell division, synaptic transmission, STAT3 signaling pathways, TGFB pathways, autophagy process, import into cell, response to ketone, and some cellular responses such as to interleukin-1 and stress, etc. (Fig. 4a). Interestingly, target mRNA enrichments in DisGeNET identified Neurodevelopmental Disorders and Neuropathy in the very top few enriched clusters (Fig. 4b), indicating the importance of those mRNAs in neurodisease.

## Discussion

The prognosis of profound SSNHL is unpredictable and poor, with complete recovery, partial recovery, or no improvement on hearing may occur. Even with the recommended steroid therapy, more than half of the patients obtain no improvement in hearing loss [4, 5]. Factors impacting hearing recovery include age at onset of hearing loss, hearing loss severity and frequencies affected, presence of vertigo, and time between onset of hearing loss and visit with the treating physician. Consistently, our study indicates that even an average of half a day earlier for medical attention after symptom onset might improve outcomes. Our study achieved a recovery rate of 19%, higher than that of 8% in previous studies [4]. We hypothesized that the combined antifibrinolytic therapy, neurotrophic support, and meticulous care during hospitalization might be beneficial, which warrants further research. But about 44% no improvement in this study (14 out of 32), still verified the worst prognosis of the profound SSNHL and unpredictable treatment response.

A few studies identified differentially expressed miRNAs from serum [19], plasma [20, 21] that are related to hearing loss under noise exposure or blood pressure conditions. Recently, serum-derived exosomes’ miRNAs have been identified for investigating potential SSNHL disease markers from mainly enrolled mixed or non-profound cases [12, 22–25], but few study has specifically focused on the plasma derived exosomes from worst prognostic profound SSNHL subtypes. Chen et al. [13] identified 66 differential expressed miRNAs in plasma EVs, but they do not disclose the SSNHL subtypes, and the sample size were even smaller (3 patients), preventing a direct molecular comparison with our findings for the profound SSNHL subtypes. Amongst those 66 miRNAs, the down regulated hsa-miR-532-3p also identified in our study. The rest candidate miRNAs in our exploratory study ( 2 upregulated and 9 downregulated miRNAs) have not yet been identified in other studies. One reason might be due to the distinct biological origins of circulating exosomes in serum or plasma, which carry different molecular fingerprints. Most studies used serum exosome fractions that are usually considered to be contaminated with CD41⁺/CD61⁺ platelet EVs and their associated miRNAs/proteins, diluting or masking disease-specific signals. Very few studies used plasma-derived exosome that are the preferred source for disease-marker identification [26]. The second reason might lie in the complex pathogenesis of the SSNHL and some potential confounding factors were not fully controlled in this study. The last but not least reason might be the limitation of the small sample size and thereof the statistics. The false-positive findings might result from nominal p values without FDR correction in this study. Whether the 12 miRNAs are specific for profound SSNHL or some of them are a genetic response to certain etiologic factors warrants a larger cohort study to verify in the future.

Many efforts have been taken to discern prognostic factors for profound SSNHL patients, but none are yet routinely available in clinical practice [3]. For example, complement C3 was identified as a severity and early-steroid-response marker [13]. Exosome miRNAs can change the fate of recipient cells by autocrine and paracrine signaling. For example, exosomal miRNAs are reported to be involved in core pathological processes of SSNHL, such as processes of hair cell damage and spiral neuron degeneration [6, 27]. It is therefore respectful that we explore the prognosis of profound SSNHL with some possible exosome miRNAs. Amongst our 14 hub candidate exsomal miRNAs, hsa-miR-132-3p and hsa-miR-146a-3p have been identified as disease marker for mixed SSNHL [13, 23], and the miR-183 family members have been reported to play a key role in the development of inner ear hair cells [28] and development of the auditory hindbrain [29]. But none of them are explored with severity of the profound SSNHL. In our study, some candidate miRNAs expression showed unique or even stronger dysregulation when stratifying the patients according to prognosis. For example, compared to similar levels in the complete recovery (BR) group with the healthy hearing group, hsa-miR-320d decreased to an undetectable level while hsa-miR-146a-3p showed a significant increase in the no improvement (BN) group. Similarly, 219a-2-3p showed unique upregulation in the no improvement group, while hsa-miR-6842-3p showed unique downregulation in the complete recovery group. It will be a valuable study to verify those candidate miRNAs in another retrospective cohort of large samples and their expression relation to target genes, such as complement C3, in the future, and do experimental exploration in the cochlear inner hair cell or sensory cell regeneration.

Understanding the complex interplay between miRNAs and their target mRNAs could pave the way for novel therapeutic strategies aimed at improving treatment outcomes in SSNHL patients. The target mRNAs of the top 6 candidate DEE miRNAs enriched in important biological processes such as autophagy, synaptic transmission, neuronal survial and responses to stress and inflammation etc., further supporting their close relation to the clinic applications. Similarly, microRNAs in pro-apoptotic and autophagy pathways are reported in presbycusis [30]. Findings from enrichment in ketone response and those from laboratory abnormal metabolic tendency may potentially echo each other, albeit the study still needs a larger cohort to reach a conclusion. Future studies should focus on validating these miRNAs and their target mRNAs in larger cohorts, exploring their potential as biomarkers and therapeutic targets for SSNHL, and experimental verification for the interaction between miRNA and target genes in certain recipient cells.

To conclude, our pilot study using small samples of the particular profound subgroup of SSNHL and stratified patients by hearing recovery degree identified possible DEE miRNAs that may serve as candidate biomarkers or prognosis predictors for the profound subtype of SSNHL. But our study has several limitations. First and the most, the research cohort is very small. This very small cohort inevitably reduces statistical power and inflates the risk of both false-positive and false-negative results. For the purpose of exploring a broad set of candidate microRNAs, the P-value-based analysis other than false-discovery-rate (FDR) adjustment was performed for statistical analysis, which might further increase false-positive findings. Consequently, our findings must be viewed as hypothesis-generating rather than definitive. Second, the study lacks validation of DEE miRNAs in other cohorts, which might narrow the candidate list. The independent replication or validation in external set of profound-SSNHL patients is needed to see the generalizability of the candidate miRNAs. Third, this pilot study lacks of longitudinal data. We compared pre-treatment exosomal profiles but did not examine post-treatment changes; thus we cannot distinguish miRNAs that respond to therapy from those that truly predict baseline prognosis. In the next study, we will recruit a larger research cohort and perform a more rational study design.

## Conclusions

Our study explored the differential expression of miRNAs in profound SSNHL and across prognostic subgroups. The findings highlight the importance of further investigating these miRNAs and their target mRNAs to develop novel therapeutic strategies for profound SSNHL, the worst and unpredictable subtype of SSNHL. The findings suggest that miRNAs—hsa-miR-320d, hsa-miR-146a-3p, hsa-miR-132-3p, hsa-miR-9-3p, hsa-miR-219b-5p, and hsa-miR-219a-2-3p may modulate key pathways involved in autophagy, ketone response, STAT3 signaling and neuronal transmission processes.

## Supplementary Information

Supplementary Material 1

## Data Availability

The datasets generated and/or analysed during the current study have been deposited in NCBI SRA repository ( [https://www.ncbi.nlm.nih.gov/bioproject/](https://www.ncbi.nlm.nih.gov/bioproject) ) with the primary accession code PRJNA1347127.

## Abbreviations

SSNHL: Sudden sensorineural hearing loss
miRNAs: MicroRNAs
DEEMs: Differentially expressed exosome miRNAs
EV: Extracellular-vesicle
PTA: Pure-tone audiometry
ABR: Auditory brain-stem response
ASSR: Auditory steady-state response
DPOAEs: Distortion-product otoacoustic emissions
TPM: Transcript per million
GO: Gene Ontology and
KEGG: Kyoto Encyclopedia of Genes and Genomes
BT: Before treatment
BR: Recovered from samples before treatment
BN: No improvement from the samples before treatment
HC: Healthy hearing controls
