# Supplementary material for: Differentially expressed exosome miRNA profiles as putative prognostic biomarkers for profound sudden sensorineural hearing loss
Source: BMC Med Genomics. 2026 Feb 24;19:56. doi: 10.1186/s12920-026-02330-9 (PMC13049808; doi:10.1186/s12920-026-02330-9)
Supplement: Supplementary file 1 — Supplementary Material 1 [file 12920_2026_2330_MOESM1_ESM.docx]

Table 1 Baseline characteristics of patients and health control group

| Characteristics | Patients  N = 12 （%） | Health  N =6 （%） | *P* |
| --- | --- | --- | --- |
| Age (year) | 48.33 ± 14.73 | 47.50 $\pm16$.40 | 0.918 |
| Gender  Male | 6 (50.00%) | 3 (50.00%) | 1.000 |
| Coexisting medical conditions |  |  |  |
| Diabetes | 1 (8.33%) | 0 (-) | 1.000 |
| Hypertension | 4 (33.33%) | 2 (33.33%) | 1.000 |
| Cardiovascular disease | 3 (25%) | 0 (-) | 0.515 |
| Head and neck tumors | 6 (50.00%) | 3 (50.00%) | 1.000 |
| Other chronic diseases | 8 (66.67%) | 6 (100.00%) | 0.245 |
| Signs and symptoms |  |  |  |
| Tinnitus | 9 (75%) | 0 (-) | 0.009 |
| Vertigo or dizziness | 9 (75%) | 0 (-) | 0.009 |
| Ear fullness or pressure sensation | 9 (75%) | 0 (-) | 0.009 |
| Emergency | 8 (66.67%) | 0 (-) | 0.013 |
| Diseased ear | 12 (100%) | 0 (-) | < 0.001 |
| Cervical spondylosis | 1 (8.33%) | 0 (-) | 1.000 |

Table 2 Laboratory findings of patients on admission to hospital

| Characteristics | Patients  N = 12 （%） | Health  N =6 （%） | *P* |
| --- | --- | --- | --- |
| Blood routine |  |  |  |
| WBC 3.5-9.5 (10^9^/L) | 9.41 ± 3.33 | 6.72 ± 1.65 | 0.091 |
| Neutrophil 40-75 (%) | 63 ± 10 | 58 ± 13 | 0.459 |
| Neutrophil# 1.8-6.3 (10^9^/L) | 6.01 ± 2.71 | 3.90 ± 1.28 | 0.100 |
| Lymphocyte 20-50 (%) | 29 ± 9 | 33 ± 12 | 0.465 |
| Lymphocyte# 1.1-3.2 (10^9^/L) | 2.66 ± 0.89 | 2.24 ± 1.05 | 0.424 |
| Monocyte 3-10 (%) | 5.84 ± 1.07 | 5.63 ± 0.80 | 0.687 |
| Monocyte# 0.1-0.6 (10^9^/L) | 0.53 ± 0.16 | 0.38 ± 0.12 | 0.073 |
| Eosinophilic 0.4-8.0 (%) | 1.30 (0.20-2.40) | 2.45 (0.90-3.00) | 0.517 |
| Eosinophilic# 0.02-0.52 (10^9^/L) | 0.13 (0.02-0.23) | 0.12 (0.06-0.21) | 0.723 |
| Basophil 0-1 (%) | 1.30 (0.20-2.40) | 2.45 (0.90-3.00) | 0.517 |
| Basophil# 0-0.06 (10^9^/L) | 0.04 ± 0.03 | 0.05 ± 0.02 | 0.674 |
| RBC 3.8-5.1 (10^12^/L) | 0.04 (0.02-0.05) | 0.05 (0.03-0.06) | 0.514 |
| Haemoglobin 115-150 (g/L) | 132 ± 16 | 134 ± 18 | 0.833 |
| MCV 82-100 (fL) | 87 ± 3 | 93 ± 4 | 0.007 |
| MCH 27-34 (pg) | 29.61 ± 1.53 | 30.87 ± 1.25 | 0.118 |
| MCHC 316-354 (g/L) | 340 ± 11 | 332 ± 8 | 0.146 |
| PLT 125-350 (10^9^/L) | 240 ± 75 | 234 ± 34 | 0.848 |
| PCT 0.114-0.282 (%) | 0.27 ± 0.04 | 0.24 ± 0.04 | 0.131 |
| MPV 6.8-11.8 (fL) | 10.67 ± 0.68 | 10.28 ± 0.54 | 0.270 |
| PDW 9.8-17.2 (fL) | 12.30 ± 1.31 | 11.73 ± 1.66 | 0.489 |
| P-LCR 13-43 (%) | 30 ± 6 | 27 ± 5 | 0.323 |
| Hematocrit 0.35-0.45 | 0.39 ± 0.04 | 0.40 ± 0.05 | 0.554 |
| RDW 39-46 (fL) | 40 ± 2 | 43 ± 4 | 0.056 |
| RDW-CV 10.1-16 (%) | 12.58 ± 0.54 | 12.47 ± 0.71 | 0.738 |
| Coagulation profile |  |  |  |
| PT-sec 9.4-12.5 (sec) | 11.51 ± 0.88 | 11.08 ± 0.43 | 0.284 |
| PT-R 80-150% | 105 ± 13 | 98 ± 6 | 0.232 |
| PT-INR 0.8-1.2 (INR) | 1.07 ± 0.08 | 1.02 ± 0.04 | 0.156 |
| APTT 26.0-41.0 (sec) | 29 ± 5 | 32 ± 3 | 0.170 |
| TT 12-17.5 (sec) | 15.91 ± 2.61 | 15.25 ± 1.71 | 0.585 |
| Fibrinogen 2.0-4.0 (g/L) | 2.43 ± 0.73 | 2.72 ± 0.37 | 0.383 |
| D-dimer 0-0.232 (μg/L) | 0.08 ± 0.03 | 0.09 ± 0.05 | 0.796 |
| ATIII | 103 (99-107) | 112 (112-112) | 0.558 |
| Plasminogen activity | 88 (82-102) | 89 (89-89) | 1.000 |
| vWF | 104 (96-105) | 109 (109-109) | 0.558 |
| Factor VIII | 104.80 (100.80-113.30) | 104.80 (104.80-104.80) | 1.000 |
| FDP | 0.29 (0.16-0.75) | 1.06 (0.59-1.13) | 0.052 |
| Liver function |  |  |  |
| TP 65-85(g/L) | 70 ± 4 | 65 ± 4 | 0.043 |
| Albumin 40-55(g/L) | 44 ± 3 | 42 ± 4 | 0.371 |
| Globumin 20-40(g/L) | 26 ± 3 | 23 ± 4 | 0.137 |
| Prealbumin 180-350 (mg/L) | 245.90 ± 53.84 | 250.20 ± 38.64 | 0.870 |
| A/G 1.2--2.4 | 1.73 ± 0.29 | 1.93 ± 0.50 | 0.343 |
| TBiL ≤ 21 (μmol/L) | 9.94 ± 4.04 | 8.45 ± 2.39 | 0.433 |
| DBiL ≤ 8 (μmol/L) | 3.88 ± 1.07 | 3.62 ± 0.84 | 0.626 |
| IBiL 3.0-16.0 (μmol/L) | 6.07 ± 3.09 | 4.83 ± 1.62 | 0.389 |
| Urea 2.6-7.5 (mmol/L) | 4.88 ± 1.34 | 6.60 ± 1.07 | 0.021 |
| ALT 7-40 (U/L) | 16.71 ± 6.95 | 15.48 ± 4.82 | 0.714 |
| AST 13-35 (U/L) | 14.89 ± 4.29 | 16.28 ± 5.36 | 0.586 |
| ALP 50-135 (U/L) | 59 ± 15 | 73 ± 24 | 0.202 |
| GGT 7-45 (U/L) | 21.78 ± 12.94 | 22.33 ± 13.22 | 0.937 |
| TBA 0-12 (μmol/L) | 2.40 (1.20-5.10) | 2.60 (1.30-4.20) | 0.860 |
| Cys-C 0.53-1.02 (mg/L) | 0.90 ± 0.21 | 0.92 ± 0.10 | 0.845 |
| LDH 120-250 (IU/L) | 159 ± 27 | 166 ± 44 | 0.715 |
| CK 40-200 (IU/L) | 76 ± 38 | 96 ± 48 | 0.380 |
| Glucose 3.89-6.11 (mmol/L) | 5.55 ± 1.06 | 4.63 ± 0.54 | 0.074 |
| TC 3.10-5.17 (mmol/L) | 4.53 ± 1.20 | 4.63 ± 0.53 | 0.850 |
| TG 0.40-1.71 (mmol/L) | 1.51 ± 0.95 | 1.28 ± 0.52 | 0.599 |
| HDL-c 1.29-1.55 (mmol/L) | 1.30 ± 0.32 | 1.31 ± 0.47 | 0.933 |
| LDL-c 0.00-3.36 (mmol/L) | 2.71 ± 1.08 | 2.91 ± 0.52 | 0.691 |
| sdLDL 0.243-1.109 (mmol/L) | 0.60 (0.39-1.03) | 0.92 (0.86-1.22) | 0.317 |
| Potassium 3.5-5.3 (mmol/L) | 3.94 ± 0.24 | 3.98 ± 0.29 | 0.789 |
| Sodium 137-147 (mmol/L) | 140.80 ± 1.97 | 140.70 ± 1.81 | 0.888 |
| Chlorine 99-110 (mmol/L) | 105.40 ± 1.83 | 104.90 ± 2.45 | 0.670 |
| Calcium 2.11-2.52 (mmol/L) | 2.34 ± 0.08 | 2.26 ± 0.11 | 0.108 |
| Phosphorus 0.85-1.51 (mmol/L) | 1.27 ± 0.16 | 1.30 ± 0.28 | 0.809 |
| eGFR ml/min/1.73m^2^ | 109 ± 14 | 95 ± 12 | 0.104 |
| UA 150-350 (μmol/L) | 261 ± 61 | 365 ± 64 | 0.007 |
| Creatinine 41-73 (μmol/L) | 63 ± 8 | 73 ± 10 | 0.076 |
| CO_2_CP 19-31 (mmol/L) | 24.62 ± 1.99 | 21.95 ± 2.04 | 0.026 |
| CRP ≤ 8 (mg/L) | 1.00 (0.82-3.00) | 0.50 (0.50-0.50) | 0.082 |
| Others |  |  |  |
| HBsAb 0-10 (MIU/ml) | 58.47 (1.63-96.74) | 14.05 (0.15-365.23) | 0.768 |
| HBeAg 0-1 (S/CO) | 0.39 ± 0.04 | 0.31 ± 0.10 | 0.088 |
| HBeAb ≥ 1.0 (S/CO) | 1.53 (1.12-1.92) | 1.56 (0.53-1.65) | 0.723 |
| HBcAb 0-1.0 (S/CO) | 0.11 (0.08-2.47) | 0.26 (0.06-5.86) | 0.375 |
| anti-HCV 0-1 (S/CO) | 0.06 ± 0.02 | 0.05 ± 0.01 | 0.283 |
| HIV (Ag/Ab) 0-1 (S/CO) | 0.08 (0.07-0.12) | 0.17 (0.10-0.31) | 0.069 |
| anti-TP 0-1.00 (S/CO) | 0.05 (0.02-0.08) | 0.03 (0.03-0.05) | 0.335 |
| Lupus anticoagulant dRVVT screening test | 32.10 (31.20-32.30) | 35.70 (35.70-35.70) | 0.558 |
| Lupus anticoagulant dRVVT confirmatory test | 28.80 (26.00-29.50) | 29.30 (29.30-29.30) | 1.000 |
| Standardized rates of lupus anticoagulant dRVVT | 1.00 (0.96-1.06) | 1.08 (1.08-1.08) | 0.558 |
| SCT screening test for lupus anticoagulant | 33.20 (33.00-34.20) | 39.10 (39.10-39.10) | 0.558 |
| SCT confirmatory test for lupus anticoagulant | 33.50 (32.90-36.40) | 36.80 (36.80-36.80) | 0.558 |
| Standardized rate of SCT for lupus anticoagulant | 0.88 (0.85-0.92) | 0.91 (0.91-0.91) | 1.000 |

Abbreviations: WBC, white blood cell; RBC, red blood cell; MCV, mean corpuscular volume; MCH, mean corpuscular hemoglobin；MCHC, mean corpuscular hemoglobin concentration; PLT, platelet count; PCT, platelet cubic measure distributing; MPV, mean platelet volume; PDW, platelet distribution width; P-LCR, platelet-large cell ratio; RDW, red blood cell distribution width; PT, prothrombin time; PT-R, prothrombin activity; INR, international normalized ratio; APTT, activated partial thromboplastin time; TT, thrombin time; ATIII, Antithrombin III; vWF, von Willebrand factor; FDP, fibrinogen degradation products; TP, total protein; TBiL, total bilirubin; DBiL, direct bilirubin; IBiL, indirect bilirubin; ALT, alanine aminotransferase; AST, aspartate aminotransferase; ALP, alkaline phosphatase; GGT, γ-glutamyl transferase; TBA, total bile acid; Cys-C, Cysteine protease inhibitor C; LDH, lactate dehydrogenase; CK, creatine kinase; TC, total cholesterol; TG, triglyceride; HDL-c, high density lipoprotein cholesferol; LDL-c, low density lipoprotein cholesterol; sdLDL, small dense LDL cholesterol; eGFR, Estimate the glomerular filtration rate; UA, uric acid; CO_2_CP, carbon dioxide combining power; CRP, C-reactive protein; HBsAb, hepatitis b surface antibody; HBeAg, hepatitis B e-antigen; HBeAb, hepatitis B E-antibody; HBcAb, hepatitis b core antibody; anti-HCV, anti-hepatitis c virus antibody; HIV, human immunodeficiency virus; anti-TP, treponema pallidum antibody.

Table 3 Characteristics, treatments and outcomes of patients

| Characteristics | Recovered patients  N = 6 | No-response patients  N = 6 | *P* |
| --- | --- | --- | --- |
| Age (year) | 48.50 ± 15.92 | 46.50 $\pm18.32$ | 0.844 |
| Gender  Male | 3 (50.00%) | 3 (50.00%) | 1.000 |
| Coexisting medical conditions |  |  |  |
| Diabetes | 1 (16.67%) | 0 (-) | 1.000 |
| Hypertension | 2 (33.33%) | 2 (33.33%) | 1.000 |
| Cardiovascular disease | 1 (16.67%) | 2 (33.33%) | 1.000 |
| Head and neck tumors | 3 (50.00%) | 3 (50.00%) | 1.000 |
| Other chronic diseases | 4 (66.67%) | 4 (66.67%) | 1.000 |
| Signs and symptoms |  |  |  |
| Tinnitus | 4 (66.67%) | 5 (83.33%) | 1.000 |
| Vertigo or dizziness | 3 (50.00%) | 6 (100.00%) | 0.182 |
| Ear fullness or pressure sensation | 5 (83.33%) | 4 (66.67%) | 1.000 |
| Emergency | 3 (50.00%) | 5 (83.33%) | 0.546 |
| Diseased ear |  |  |  |
| Left | 3 (50.00%) | 3 (50.00%) | 1.000 |
| Cervical spondylosis | 0 (-) | 1 (16.67%) | 1.000 |
| Days from onset to visit | 0.50 (0-1) | 1.00 (1.00-3.00) | 0.038 |
| Days of infusion | 12.50 ± 2.81 | 13.83 ± 1.47 | 0.328 |
| Pre-treatment hearing loss | 90.33 ± 8.50 | 87.33 ± 5.16 | 0.477 |
| Post-treatment hearing loss | 30.00 ± 6.12 | 86.00 ± 6.32 | < 0.001 |
| Brain evoked potential thresholds after treatment | 36.67 ± 8.17 | 98.33 ± 4.08 | < 0.001 |

Table 4 Laboratory findings of patients

| Characteristics | Recovered patients  N = 6 | No-response patients  N = 6 | *P* |
| --- | --- | --- | --- |
| Blood routine |  |  |  |
| WBC 3.5-9.5 (10^9^/L) | 9.24 ± 4.02 | 9.76 ± 1.92 | 0.841 |
| Neutrophil 40-75 (%) | 62 ± 11 | 64 ± 8 | 0.779 |
| Neutrophil# 1.8-6.3 (10^9^/L) | 5.85 ± 3.19 | 6.35 ± 1.93 | 0.810 |
| Lymphocyte 20-50 (%) | 29.77 ± 9.62 | 28.80 ± 8.35 | 0.887 |
| Lymphocyte# 1.1-3.2 (10^9^/L) | 2.65 ± 1.12 | 2.70 ± 0.19 | 0.934 |
| Monocyte 3-10 (%) | 6.15 ± 1.15 | 5.23 ± 0.64 | 0.248 |
| Monocyte# 0.1-0.6 (10^9^/L) | 0.54 ± 0.17 | 0.52 ± 0.16 | 0.840 |
| Eosinophilic 0.4-8.0 (%) | 1.15 (0.10-3.80) | 1.80 (0.20-2.40) | 0.897 |
| Eosinophilic# 0.02-0.52 (10^9^/L) | 0.09 (0.01-0.23) | 0.14 (0.02-0.27) | 0.700 |
| Basophil 0-1 (%) | 0.30 (0.20-0.80) | 0.40 (0.40-0.70) | 0.515 |
| Basophil# 0-0.06 (10^9^/L) | 0.03 (0.01-0.08) | 0.04 (0.04-0.05) | 0.694 |
| RBC 3.8-5.1 (10^12^/L) | 4.47 ± 0.48 | 4.43 ± 0.40 | 0.901 |
| Haemoglobin 115-150 (g/L) | 134 ± 16 | 129 ± 17 | 0.670 |
| MCV 82-100 (fL) | 88 ± 3 | 85 ± 3 | 0.138 |
| MCH 27-34 (pg) | 29.93 ± 1.57 | 28.97 ± 1.48 | 0.406 |
| MCHC 316-354 (g/L) | 339 ± 13 | 342 ± 8 | 0.713 |
| PLT 125-350 (10^9^/L) | 223 ± 87 | 274 ± 29 | 0.367 |
| PCT 0.114-0.282 (%) | 0.27 ± 0.05 | 0.28 ± 0.02 | 0.606 |
| MPV 6.8-11.8 (fL) | 10.86 ± 0.58 | 10.37 ± 0.85 | 0.361 |
| PDW 9.8-17.2 (fL) | 12.58 ± 1.23 | 11.83 ± 1.59 | 0.480 |
| P-LCR 13-43 (%) | 31.50 ± 4.69 | 27.47 ± 7.20 | 0.367 |
| Hematocrit 0.35-0.45 | 0.40 ± 0.05 | 0.38 ± 0.04 | 0.566 |
| RDW 39-46 (fL) | 40 ± 2 | 39 ± 2 | 0.661 |
| RDW-CV 10.1-16 (%) | 12.42 ± 0.52 | 12.90 ± 0.52 | 0.232 |
| Coagulation profile |  |  |  |
| PT-sec 9.4-12.5 (sec) | 11.21 ± 0.63 | 11.80 ± 1.05 | 0.270 |
| PT-R 80-150% | 106 ± 10 | 104 ± 17 | 0.733 |
| PT-INR 0.8-1.2 (INR) | 1.04 ± 0.06 | 1.10 ± 0.10 | 0.235 |
| APTT 26.0-41.0 (sec) | 29.67 ± 5.36 | 28.20 ± 3.84 | 0.598 |
| TT 12-17.5 (sec) | 15.03 ± 1.47 | 16.78 ± 3.31 | 0.264 |
| Fibrinogen 2.0-4.0 (g/L) | 2.64 ± 0.65 | 2.23 ± 0.80 | 0.355 |
| D-dimer 0-0.232 (μg/L) | 0.08 ± 0.02 | 0.09 ± 0.05 | 0.563 |
| ATIII | 117 ± 28 | 103 ± 4 | 0.425 |
| Plasminogen activity | 95 ± 18 | 78 ± 31 | 0.546 |
| vWF | 142 ± 53 | 94 ± 12 | 0.194 |
| Factor VIII | 157 ± 73 | 104 ± 8 | 0.271 |
| FDP | 0.29 (0.28-0.75) | 0.24 (0.12-18.10) | 1.000 |
| Liver function |  |  |  |
| TP 65-85(g/L) | 72 ± 3 | 68 ± 4 | 0.176 |
| Albumin 40-55(g/L) | 46 ± 3 | 42 ± 1 | 0.032 |
| Globumin 20-40(g/L) | 25.70 ± 3.07 | 26.15 ± 4.26 | 0.858 |
| Prealbumin 180-350 (mg/L) | 262 ± 58 | 225 ± 48 | 0.336 |
| A/G 1.2--2.4 | 1.80 ± 0.29 | 1.65 ± 0.30 | 0.474 |
| TBiL ≤ 21 (μmol/L) | 10.04 ± 4.48 | 9.83 ± 4.10 | 0.943 |
| DBiL ≤ 8 (μmol/L) | 3.86 ± 0.86 | 3.90 ± 1.44 | 0.960 |
| IBiL 3.0-16.0 (μmol/L) | 6.18 ± 3.70 | 5.93 ± 2.68 | 0.912 |
| Urea 2.6-7.5 (mmol/L) | 5.09 ± 1.68 | 4.61 ± 0.91 | 0.623 |
| ALT 7-40 (U/L) | 21 ± 5 | 12 ± 5 | 0.032 |
| AST 13-35 (U/L) | 17 ± 5 | 13 ± 2 | 0.199 |
| ALP 50-135 (U/L) | 67 ± 12 | 50 ± 14 | 0.099 |
| GGT 7-45 (U/L) | 22 (19-22) | 13 (11-34) | 0.268 |
| TBA 0-12 (μmol/L) | 3.20 (2.40-5.80) | 1.20 (0.85-3.25) | 0.178 |
| Cys-C 0.53-1.02 (mg/L) | 0.94 ± 0.25 | 0.85 ± 0.19 | 0.544 |
| LDH 120-250 (IU/L) | 171 ± 32 | 144 ± 5 | 0.132 |
| CK 40-200 (IU/L) | 92 ± 46 | 56 ± 12 | 0.175 |
| Glucose 3.89-6.11 (mmol/L) | 6.19 ± 1.03 | 4.74 ± 0.19 | 0.030 |
| TC 3.10-5.17 (mmol/L) | 4.79 ± 1.21 | 4.21 ± 1.29 | 0.506 |
| TG 0.40-1.71 (mmol/L) | 1.78 (0.93-2.10) | 0.97 (0.58-2.20) | 0.713 |
| HDL-c 1.29-1.55 (mmol/L) | 1.28 ± 0.40 | 1.32 ± 0.24 | 0.866 |
| LDL-c 0.00-3.36 (mmol/L) | 2.99 ± 1.22 | 2.37 ± 0.91 | 0.425 |
| sdLDL 0.243-1.109 (mmol/L) | 1.03 (0.40-1.60) | 0.50 (0.36-0.71) | 0.270 |
| Potassium 3.5-5.3 (mmol/L) | 4.05 ± 0.27 | 3.80 ± 0.11 | 0.121 |
| Sodium 137-147 (mmol/L) | 142 ± 1 | 140 ± 2 | 0.149 |
| Chlorine 99-110 (mmol/L) | 106 ± 2 | 104 ± 1 | 0.083 |
| Calcium 2.11-2.52 (mmol/L) | 2.38 ± 0.07 | 2.29 ± 0.05 | 0.047 |
| Phosphorus 0.85-1.51 (mmol/L) | 1.21 ± 0.18 | 1.36 ± 0.08 | 0.180 |
| eGFR ml/min/1.73m^2^ | 110 ± 16 | 106 ± 15 | 0.737 |
| UA 150-350 (μmol/L) | 274 ± 60 | 244 ± 67 | 0.502 |
| Creatinine 41-73 (μmol/L) | 61 ± 6 | 66 ± 11 | 0.467 |
| CO_2_CP 19-31 (mmol/L) | 24.90 ± 2.29 | 24.28 ± 1.83 | 0.671 |
| CRP ≤ 8 (mg/L) | 1.00 (0.63-2.00) | 1.00 (1.00-6.00) | 0.541 |
| Others |  |  |  |
| HBsAb 0-10 (MIU/ml) | 16.44 (0.77-86.89) | 84.83 (58.47-96.74) | 0.268 |
| HBeAg 0-1 (S/CO) | 0.38 ± 0.02 | 0.41 ± 0.05 | 0.286 |
| HBeAb ≥ 1.0 (S/CO) | 1.70 (0.77-1.91) | 1.33 (1.12-1.92) | 1.000 |
| HBcAb 0-1.0 (S/CO) | 0.10 (0.09-5.05) | 0.53 (0.08-2.47) | 1.000 |
| anti-HCV 0-1 (S/CO) | 0.06 ± 0.01 | 0.07 ± 0.03 | 0.655 |
| HIV (Ag/Ab) 0-1 (S/CO) | 0.12 (0.11-0.12) | 0.08 (0.06-0.08) | 0.198 |
| anti-TP 0-1.00 (S/CO) | 0.06 (0.04-0.08) | 0.04 (0.02-0.29) | 0.644 |
| Lupus anticoagulant dRVVT screening test | 29.70 ± 2.12 | 34.67 ± 4.27 | 0.238 |
| Lupus anticoagulant dRVVT confirmatory test | 25.95 ± 0.07 | 29.97 ± 1.46 | 0.034 |
| Standardized rates of lupus anticoagulant dRVVT | 1.01 ± 0.07 | 1.03 ± 0.11 | 0.867 |
| SCT screening test for lupus anticoagulant | 31 ± 3 | 37 ± 6 | 0.290 |
| SCT confirmatory test for lupus anticoagulant | 30 ± 5 | 36 ± 2 | 0.175 |
| Standardized rate of SCT for lupus anticoagulant | 0.89 ± 0.05 | 0.91 ± 0.12 | 0.786 |

Abbreviations: WBC, white blood cell; RBC, red blood cell; MCV, mean corpuscular volume; MCH, mean corpuscular hemoglobin；MCHC, mean corpuscular hemoglobin concentration; PLT, platelet count; PCT, platelet cubic measure distributing; MPV, mean platelet volume; PDW, platelet distribution width; P-LCR, platelet-large cell ratio; RDW, red blood cell distribution width; PT, prothrombin time; PT-R, prothrombin activity; INR, international normalized ratio; APTT, activated partial thromboplastin time; TT, thrombin time; ATIII, Antithrombin III; vWF, von Willebrand factor; FDP, fibrinogen degradation products; TP, total protein; TBiL, total bilirubin; DBiL, direct bilirubin; IBiL, indirect bilirubin; ALT, alanine aminotransferase; AST, aspartate aminotransferase; ALP, alkaline phosphatase; GGT, γ-glutamyl transferase; TBA, total bile acid; Cys-C, Cysteine protease inhibitor C; LDH, lactate dehydrogenase; CK, creatine kinase; TC, total cholesterol; TG, triglyceride; HDL-c, high density lipoprotein cholesferol; LDL-c, low density lipoprotein cholesterol; sdLDL, small dense LDL cholesterol; eGFR, Estimate the glomerular filtration rate; UA, uric acid; CO_2_CP, carbon dioxide combining power; CRP, C-reactive protein; HBsAb, hepatitis b surface antibody; HBeAg, hepatitis B e-antigen; HBeAb, hepatitis B E-antibody; HBcAb, hepatitis b core antibody; anti-HCV, anti-hepatitis c virus antibody; HIV, human immunodeficiency virus; anti-TP, treponema pallidum antibody.
